# Neonatal Seizure Models to Study Epileptogenesis

**DOI:** 10.3389/fphar.2018.00385

**Published:** 2018-04-18

**Authors:** Yuka Kasahara, Yuji Ikegaya, Ryuta Koyama

**Affiliations:** Laboratory of Chemical Pharmacology, Graduate School of Pharmaceutical Sciences, The University of Tokyo, Tokyo, Japan

**Keywords:** neonatal seizures, epilepsy, hypoxia-ischemia, febrile seizures, bumetanide

## Abstract

Current therapeutic strategies for epilepsy include anti-epileptic drugs and surgical treatments that are mainly focused on the suppression of existing seizures rather than the occurrence of the first spontaneous seizure. These symptomatic treatments help a certain proportion of patients, but these strategies are not intended to clarify the cellular and molecular mechanisms underlying the primary process of epilepsy development, i.e., epileptogenesis. Epileptogenic changes include reorganization of neural and glial circuits, resulting in the formation of an epileptogenic focus. To achieve the goal of developing “anti-epileptogenic” drugs, we need to clarify the step-by-step mechanisms underlying epileptogenesis for patients whose seizures are not controllable with existing “anti-epileptic” drugs. Epileptogenesis has been studied using animal models of neonatal seizures because such models are useful for studying the latent period before the occurrence of spontaneous seizures and the lowering of the seizure threshold. Further, neonatal seizure models are generally easy to handle and can be applied for *in vitro* studies because cells in the neonatal brain are suitable for culture. Here, we review two animal models of neonatal seizures for studying epileptogenesis and discuss their features, specifically focusing on hypoxia-ischemia (HI)-induced seizures and febrile seizures (FSs). Studying these models will contribute to identifying the potential therapeutic targets and biomarkers of epileptogenesis.

## Introduction

The neonatal period is at higher risk of having seizures than other periods in life (Annegers et al., 1995). A population study indicated the incidence of seizures to be 1–5 per 1000 live births (Saliba, 2001; Volpe, 2008; Glass, 2014). Early life insults such as hypoxic-ischemic encephalopathy and fever are major causes of neonatal seizures (Rakhade and Jensen, 2009), and it has been reported that 16–56% of neonates that experience seizures develop epilepsy later in life (Mizrahi and Watanabe, 2005; Pisani et al., 2012). The primary process whereby principal neurons generate the first spontaneous and epileptiform discharges is referred to as epileptogenesis and is often accompanied by both structural and functional alteration of neuronal circuits (Goldberg and Coulter, 2013). Classical anticonvulsants, i.e., anti-epileptic drugs, are used to suppress ongoing and future seizures but not to prevent the onset of epilepsy (Temkin, 2009). Therapeutic strategies targeting the prevention of epileptogenesis are expected to prevent the onset of epilepsy, i.e., the occurrence of the first spontaneous epileptic seizures. To develop such “anti-epileptogenic” treatments, the cellular and molecular mechanisms underlying epileptogenesis have been studied using animal models of neonatal seizures (Dubé et al., 2006, 2010; Kadam et al., 2010). This mini-review will provide an overview of the representative animal models of neonatal seizures, especially focusing on rodent models of hypoxia-ischemia (HI)-induced seizures and febrile seizures (FSs) (Dubé et al., 2010; Sun et al., 2016). We will also discuss potential pharmacological strategies that could target epileptogenic changes observed in neonatal seizure models.

## Hypoxia-Ischemia (HI) Models

Neonatal seizures are often induced by perinatal asphyxia with a critical lack of oxygen during labor and delivery (Sun et al., 2016). To study this type of seizure and its sequelae, experimental HI-induced seizures have been extensively used. In this section, we will introduce rodent models of HI that are potentially useful to identify mechanisms that mediate epileptogenesis in the immature brain. Rice et al. (1981) first established a rat neonatal HI model known as the Rice–Vannucci model. The Rice–Vannucci model and its derivatives have been widely used to study neonatal HI. For preparing the HI model, rat pups of both sexes are typically used at postnatal day 7 (P7)-P10 because the brain development during this period roughly corresponds to that of humans in late embryonic to early postnatal periods (Talos et al., 2006; Rakhade and Jensen, 2009). It should be noted that sex differences in susceptibility to HI have been reported (Smith et al., 2014a). To induce HI, rat pups are exposed to a hypoxic environment (8% O_2_ in N_2_) for 30 min–2.5 h (sometimes up to 4 h) after ligation of the lateral common carotid artery. In this system, brain damage, including selective neuronal necrosis and infarction in the ipsilateral cerebral cortex, striatum, and hippocampus, was observed in no less than 90% of HI-induced animals and within 50 h after HI induction (Rice et al., 1981). In addition, the authors revealed that lower O_2_ levels and longer exposure time to HI resulted in more-severe brain damage (Rice et al., 1981; Cuaycong et al., 2011). In HI models, cortical damage is mainly observed in columns at right angles to the pial surface, a pattern that is also observed in human infants who experienced repeated hypoxia-acidosis with hypotension (Norman, 1981; Rice et al., 1981). HI models also replicate many histological features, such as porencephalic cysts and cortical microgyri, observed in brains of infants with hypoxic-ischemic encephalopathy (Williams et al., 2004; Kadam and Dudek, 2007; Williams and Dudek, 2007). Further, 90 min of HI treatment evoked cortical electrographic seizures with correlated behavioral movements in 92% (11 of 12) and electrographic seizures in 83% (10 of 12) of the rats (Cuaycong et al., 2011). Additionally, simultaneous video monitoring and electroencephalogram (EEG) recording during and after HI induction revealed that all rats (12 of 12) showed electroclinical seizures, defined as EEG patterns abnormal in amplitude and frequency, and that the seizures persist for 24 h in 67% (8 of 12) and 48 h in 25% (3 of 12) (Sampath et al., 2014).

## Seizure Phenotypes Following HI Induction

Kadam et al. (2010) examined whether HI insults initiate epileptogenic processes. Rat pups at P7 underwent unilateral carotid ligation followed by a hypoxic environment (8% O_2_, 2 h), and continuous radio-telemetry and video recording of cortical electroencephalographic and behavioral seizures were performed from 2 to 12 months of age. It was reported that 56% (10 of 18) of HI-induced rats develop spontaneous epileptiform discharges and recurrent seizures. Furthermore, all rats with spontaneous electrographic seizures exhibited obvious cystic infarcts in the ipsilateral hemisphere, although the seizure rate per day did not always depend on the severity of infarct. Rats without spontaneous seizures exhibited no infarct and no associated neuronal death. It was suggested that the occurrence of cortical abnormalities is associated with the acquisition of epileptogenesis, i.e., the occurrence of first spontaneous seizures. Spontaneous electrographic seizures, which are often accompanied by behavioral seizures, occurred in distinct clusters with seizure-free periods as long as a few weeks and progressively became more severe and more frequent over time. In addition, 24-h behavioral monitoring for a week/month in HI-induced rats starting from P30 revealed that behavioral seizures were not detected until the second month of monitoring (Kadam and Dudek, 2007). These results indicate that the process of epileptogenesis requires a long and continuous latency period, which may allow therapeutic intervention to prevent subsequent progression of the epileptogenic process, especially by suppressing structural brain damage.

Some studies, however, reported few or no incidence of spontaneous seizures following HI (**Table 1**). When mouse pups were subjected to HI induction at P7 with a ligation of the lateral common carotid artery and a subsequent hypoxic challenge (8% O_2_ for 45 min), evident epileptiform discharges were not observed at 2–3 months of age during hippocampal and cortical EEG recordings performed 5–8 h/day for 3–4 days (Peng et al., 2015). Chronic video monitoring for 10 consecutive days revealed that motor seizures occurred in only 2 of 23 post-HI mice. In addition, to evaluate seizure susceptibility of HI-induced mice, the authors stimulated the hippocampus with square current pulses to evoke motor seizures and found no evidence of increased seizure susceptibility in HI mice. These conflicting data in terms of reproducing HI-induced development of epilepsy may be attributable to the differences in animal species, sex (both sexes are often mixed), hypoxic environment (especially the duration of hypoxia), seizure criteria, and EEG recording time, as well as to the small numbers of animals in control groups in some cases. The establishment of stable HI models using mice is awaited because transgenic lines are more available in mice than in rats and will enable the study of cellular and molecular mechanisms underlying epileptogenesis.

**Table 1:** Epileptogenesis in neonatal seizure models.

Model	Animal species/strain	Age	Behavioral seizures during induction	Electrographic seizures during induction	Spontaneous seizures	Reference
HI	Sprague–Dawley rats	P30	N/A	N/A	15% (3 of 20)	Williams and Dudek, 2007
HI	Sprague–Dawley rats	P7	N/A	N/A	30% (3 0f 10)	Kadam and Dudek, 2007
HI	Sprague–Dawley rats	P7	N/A	N/A	56% (10 of 18)	Kadam et al., 2010
Hypoxia	Long-Evans rats	P10	93% (58 of 61)	Yes	94% (48 of 51)	Rakhade et al., 2011
FS	Sprague–Dawley rats	P10-11	Yes	N/A	35% (6 of 17)	Dubé et al., 2006
FS	Sprague–Dawley rats	P11	Yes	N/A	50% (8 of 16)	Koyama et al., 2012
Kainic acid	c-Dawley rats	P5-60	Yes	Yes	N/A (KA at P5, P10) 14% (P20) 30% (P30) 44% (P60)	Stafstrom et al., 1992
Flurothyl	Sprague–Dawley rats	P0-9	Yes	N/A	Increased seizure susceptibility to flurothyl	Huang et al., 1999

*HI, hypoxia-ischemia; FS, febrile seizure*.

## Febrile Seizure (FS) Models

Fever (typically greater than 38°C)-induced FSs are the most common childhood seizures, with a prevalence of 2-14% worldwide (Verity and Golding, 1991; Vestergaard and Christensen, 2009; Koyama and Matsuki, 2010). FSs usually occur between 3 months and 5 years of age with the peak at 16–18 months. Although simple FSs are mostly benign, complex FSs with prolonged duration (>15 min), recurrent seizures or focal neurological features are associated with the development of temporal lobe epilepsy (Cendes et al., 1993; French et al., 1993; Theodore et al., 1999). To investigate the potential consequences of complex FSs and to screen potential anti-epileptogenic drugs, various highly reproducible animal models have been developed (Koyama, 2017). In these models, prolonged seizures are induced by exposing rodent pups to a hyperthermic environment to simulate fever-like conditions; the main such models are the hair dryer model (Dubé et al., 2000; Koyama et al., 2012; Tao et al., 2016), heated chamber model (Holtzman et al., 1981; Schuchmann et al., 2006), hot water model (Ullal et al., 1996; Jiang et al., 1999), microwave model (Hjeresen et al., 1983), and lipopolysaccharide model (Heida et al., 2004). Although there are limitations to completely mimicking clinical phenotypes of FSs in human neonates, experimental hyperthermia can induce the release of fever mediators that are necessary for the onset of FSs (Dubé et al., 2005).

Baram and his colleagues developed and refined the hair dryer model that is widely used (Toth et al., 1998; Dubé et al., 2010). In this model, P10–11 Sprague–Dawley rats or P14–15 129/Sv or C57BL/6 mice are often used because the developmental stage of hippocampus is roughly comparable to that in human infants, which are most susceptible to FSs. Significant sex differences were not observed, at least for the seizure duration (Lemmens et al., 2005). It has been reported that differences in mouse strain affect susceptibility to FS induction (van Gassen et al., 2008). Prolonged FSs are generated by maintaining hyperthermia (typically 38.5–42.5°C) for 30 min. During hyperthermia, the rectal temperature is measured every 2 min to maintain core temperature between 38.5 and 42.5°C, which corresponds to the threshold temperature to evoke complex FSs in human neonates. Hyperthermic controls can be conducted by administering barbiturates to suppress seizures for distinguishing whether the observed FS consequences result from the seizures or the hyperthermia. The onset of experimental FSs typically consists of an acute sudden arrest of hyperthermia-induced hyperactivity, such as oral automatism and running. Oral automatism is typically followed by forelimb clonus. In the later phase of FSs, tonic body flexion is often observed in rats, but it is rare in mice.

## Seizure Phenotypes Following FS Induction

Dubé et al. (2000) showed that FSs during development decreased the seizure threshold in later life in rats. In brief, all the rats (11 of 11) at 10–11 weeks following early life FSs developed hippocampal seizures, and most (8 of 11) experienced status epilepticus after intraperitoneal administration of a subthreshold dose of the chemical convulsant kainate (5 mg/kg). In contrast, most normothermic (6 of 8) and hyperthermic controls without seizures (5 of 6) did not exhibit seizures or kainite-induced status epilepticus. The authors prepared hippocampal-entorhinal cortical slices 1 week after FS induction and then stimulated Schaeffer collaterals, observing more prolonged status epilepticus-like discharges than in control slices. The same research group examined whether experimental FSs induce spontaneous seizures by performing chronic monitoring of behaviors and EEGs from the hippocampus and cortex (Dubé et al., 2006). 2 months after FS induction, bipolar electrodes were implanted unilaterally into the dorsal hippocampus and frontoparietal cortex. After the surgery, long-term video-EEG recordings were performed on postnatal days 90, 105, 120, 135, 165, and 180 for 5 h at night. None of the normothermic or hyperthermic controls developed spontaneous seizures or interictal events, whereas behavioral and hippocampal electrographic spontaneous seizures in 35% (6 of 17) and interictal epileptiform EEG abnormalities in 88% (15 of 17) of FS rats were observed. Moreover, longer duration of experimental FSs increased the probability of developing subsequent spontaneous seizures (Dubé et al., 2010). These results suggest that hyperthermia-induced experimental FSs lead to progression of the epileptogenic process.

## Pharmacological Strategies

Neonatal seizures should be treated adequately and rapidly because prolonged seizures would result in severe neurological morbidity (Ronen et al., 2007; Jensen, 2009). However, seizures in neonatal periods are sometimes resistant to conventional anti-epileptic drugs. For example, phenobarbital, which binds to an allosteric site on the GABA_A_ receptor and thereby potentiates the action of endogenous GABA, has been used as the first-line treatment for neonatal seizures (WHO) (World Health Organization, 2011). However, studies have reported that fewer than 50% of neonates with seizures respond to phenobarbital in terms of the suppression of electrographic seizures (Painter et al., 1999; Boylan et al., 2002, Rennie and Boylan, 2007). In addition, it has been reported that phenobarbital could induce the apoptosis of neurons in gray and white matter and impair synaptic connectivity in the immature brain (Bittigau et al., 2003; Forcelli et al., 2012; Kaushal et al., 2016). Moreover, the administration of phenobarbital is associated with long-term alterations in behavioral phenotypes, including impaired cognition and depressive behaviors (Stefovska et al., 2008; Brodie and Kwan, 2012). Thus, more efficient pharmacological treatment than the use of phenobarbital alone should be considered to enhance the inhibitory effects of GABA in neonatal periods.

Because GABA can depolarize immature neurons that overwhelm mature neurons in the developing brain, GABA-mimetic and GABA-modulating anti-epileptic drugs are relatively ineffective in neonatal seizures. It depends on the intracellular Cl^-^ levels and the Cl^-^ equilibrium potential whether GABA provokes inhibitory or excitatory actions. The intracellular Cl^-^ levels are mainly controlled by cation-chloride cotransporter (CCC) family that involves Na^+^K^+^2Cl^-^ co-transporter isoform 1 (NKCC1), which mediates Cl^-^ influx, and K^+^2Cl^-^ cotransporter isoform 2 (KCC2), which mediates Cl^-^ efflux. The balance in the expression levels of NKCC1 and KCC2 shapes the developmental changes in the actions of GABA: early expression of NKCC1 and late expression of KCC2 underlie the excitatory action of GABA in immature neurons because of an elevated intracellular Cl^-^ level and a depolarized Cl^-^ equilibrium potential. It has been suggested that the diuretic bumetanide, a selective NKCC1 inhibitor, could be useful for epileptogenic treatment (Löscher et al., 2013a,b), although it should be noted that the efficacy of bumetanide is still controversial (Puskarjov et al., 2014; Ben-Ari et al., 2016; Hernan and Holmes, 2016). It has been reported that bumetanide blocked kainate-induced seizures in neonatal rats (Dzhala et al., 2005) and that the treatment of bumetanide alone or bumetanide with phenobarbital decreased seizure events and susceptibility after early life seizures in some animal models (Cleary et al., 2013; Holmes et al., 2015; Hu et al., 2017). Koyama et al. (2012) revealed that excitatory GABA_A_ signaling mediates the emergence of ectopic granule cells that lead to hippocampal hyperexcitability and the development of epilepsy in FS rats. Continuous administration of bumetanide (0.1 mg/kg, i.p.) after FSs attenuated ectopic localization of granule cells, susceptibility to limbic seizures and development of epilepsy. In an HI model, bumetanide suppressed mossy fiber sprouting, an anatomical hallmark that may shape epileptogenic neural circuits (Koyama and Ikegaya, 2004), after hypoxia and prevented the onset of spontaneous electrographic seizures (Wang et al., 2015). In human patients, Pressler et al. (2015) conducted a trial of bumetanide for infants with hypoxic-ischemic encephalopathy, but this study was terminated due to the lack of clear efficacy and side effects of bumetanide. In clinical trials of human neonatal seizures, subjects are limited to infants with phenobarbital-resistant refractory seizures.

For the development of anti-epileptogenic drugs, future studies using various prodrugs of bumetanide or alternative NKCC1 blockers with enhanced penetration into the brain through the blood brain barrier, or drugs that enhance Cl^-^ extrusion via KCC2, are essential (Löscher et al., 2013b; Kaila et al., 2014; Puskarjov et al., 2014). The efficacy of bumetanide would depend on seizure phenotypes and the stage of epileptogenic process. Thus, it is important to clarify the changing roles of GABAergic signaling in the early and late phase of epileptogenesis. Some studies have reported the involvement of excitatory GABA_A_ signaling in rather late phase of epileptogenic changes, i.e., structural changes, after neonatal seizures (Koyama et al., 2012; Wang et al., 2015). However, the roles of excitatory GABA_A_ signaling in very early stage of epileptogenesis remain unclear. Thus, proper methods for recording the changes in neuronal activity in the onset of neonatal seizures to examine the effects of GABAergic signaling are necessary.

## Conclusion

Proper treatment of neonatal seizures is essential to prevent the future development of epilepsy. However, evidence-based guidelines for pharmacological treatment are lacking (Slaughter et al., 2013; Glass, 2014). In this mini-review, we described two representative animal models of neonatal seizures with a relatively higher risk of epilepsy later in life. These animal models successfully replicate some of the structural abnormalities and cognitive dysfunctions that have been reported in human individuals who experienced neonatal seizures (Dubé et al., 2009; Smith et al., 2014b; Tao et al., 2016). However, the findings from the experimental neonatal seizures are not always consistent.To avoid the inconsistency, it is of importance to consider the differences in experimental conditions such as animal species, sexes, developmental stages, and the criteria used to categorize seizures. It is also helpful to develop additional experimental models that mimic the initial triggers of neonatal seizures in humans, for example, exanthema subitum or influenza that lead to fever production. It should be also noted that the biomarkers to diagnose the development of epilepsy later in life need to be discovered, especially in terms of drug administration to infants and children. The proper adoption and use of animal models will allow investigators to clarify the cellular and molecular mechanisms of epileptogenesis following neonatal seizures and to identify pharmacological targets and biomarkers in human neonates.

## Author Contributions

YK and RK wrote the manuscript. YK, RK, and YI discussed the manuscript.

## Conflict of Interest Statement

The authors declare that the research was conducted in the absence of any commercial or financial relationships that could be construed as a potential conflict of interest.
